# Therapist Anxious Distress and Avoidance of Implementing Time-Out

**DOI:** 10.1007/s10578-024-01706-1

**Published:** 2024-05-31

**Authors:** Corinna C. Klein, Hanan Salem, Emily M. Becker-Haimes, Miya L. Barnett

**Affiliations:** 1 Department of Counseling, Clinical, & School Psychology, University of California, Santa Barbara, Santa Barbara, CA USA; 2 Department of Psychiatry, University of Pennsylvania Perelman School of Medicine, Philadelphia, PA USA; 3 Department of Psychiatry, University of California, Los Angeles, Los Angeles, CA USA

**Keywords:** Therapist anxiety, Mental health services, Implementation, Evidence-based intervention, Time-out

## Abstract

Therapist anxious distress when delivering child mental health treatment has been understudied as a factor that contributes to the underuse of some evidence-based interventions (EBIs), such as time-out for children with disruptive behaviors. This study investigated therapist anxious avoidance of time-out using a three-part, vignette-based survey design. Therapists (n = 198) read a vignette of an in-session time-out and reported on their personal anxious distress and likelihood of discontinuing the implementation of time-out. Therapists also provided open-ended descriptions of challenges to delivering time-out. Therapists reported moderate anxious distress at time points 1 and 2 and lower anxious distress at time 3 when the time-out had resolved. Most therapists endorsed some avoidance of time-out. Binomial logistic regression analyses indicated that increased anxious distress corresponded with an increased probability of avoiding time-out delivery in the future. Qualitative reports expanded on challenges to implementing time-out. Findings suggest the importance of addressing therapist anxious distress when implementing children’s mental health treatments.

Providing evidence-based treatment to children and families is critical for ensuring the quality and effectiveness of care. However, most therapists do not provide evidence-based treatment [1, 2]. The field of implementation science has attempted to identify factors that impact the implementation and dissemination of evidence-based practices in community mental health agencies, to ensure that clients are receiving effective care. Leading implementation science frameworks focus on contextual factors [3], organizational factors [4], intervention characteristics [5], therapist factors [6, 7], and the interaction between these factors [8]. Models focusing on therapist factors have primarily investigated therapist attitudes [9], knowledge [10], and self-efficacy [11]. However, training therapists to increase knowledge of evidence-based treatments has not been found to ensure improve client outcomes [12]. Similarly, therapist attitudes towards evidence-based practices have been found to impact their uptake and sustainment, however attitudes do not entirely account for the omission of empirically-supported treatments in care provision [13].

Therapist anxiety has been proposed as an additional factor impacting the uptake of EBIs [14, 15]. In fact, preliminary research has suggested that higher rates of therapist anxiety, measured in one study as intolerance of uncertainty, are related to decreased use of evidence-based interventions, specifically exposure-based and behavioral experiments [16]. A systematic review of therapist non-adherence to evidence-based treatments identified six studies that investigated the role of therapist anxiety in treatment delivery [17]. Identified studies focused primarily on the delivery of CBT and exposure-based or behavioral interventions and found associations between therapist anxiety and decreased or minimized use of treatment elements likely to cause temporary anxiety in patients (i.e. exposures and behavioral experiments). Therapists have been found to prefer more emotionally benign elements of treatment, worrying less about providing psychoeducation [18] and opting for relaxation strategies over exposure-based interventions [1].

The impact of therapist anxiety on EBI delivery has been discussed primarily in the context of anxiety disorders [19, 20], OCD [21] eating disorders [16, 18], and suicide prevention [22, 23]. It has not yet been investigated in the context of parent behavior therapy, the leading treatment for childhood disruptive behaviors [24], despite ongoing obstacles to community implementation of behavioral parent therapy. Teaching caregivers to use time-out for youth disruptive behaviors is one component of treatment that has been identified as a key element in treating childhood externalizing symptoms (e.g. aggression, hyperactivity, oppositionality) [25, 26]. Time-out is a non-coercive behavioral strategy involving a temporary removal of parental attention and proximity [24]. When used effectively, time-out can ameliorate parental discipline by providing consistent and predictable limits and consequences, and offering children an opportunity to develop self-regulation skills [27]. Time-out is taught within the context of a warm, nurturing relationship as a response to aggression or other conduct problems. Treatments that include time-out either offer didactic instruction on how to implement time-out at home, or coach parents to practice delivering time-out in session [28]. When delivering in-session time-outs, therapists coach caregivers to implement the strategy effectively through in-vivo coaching, which has been found to improve the uptake of new parenting skills [29].

Despite positive outcomes associated with teaching caregivers to use time-out, it is the least used component of behavioral parent training (BPT) by community therapists [30]. Therapists have described time-out as unacceptable, reported negative beliefs about the strategy, and expressed concern that it may worsen child behavior and anxiety [31]. Many recent articles have tried to understand impediments to the use of time-out in children’s mental health treatment [32–34], with some focusing on therapist perspectives [35]. No articles to date have specifically investigated the role of a therapist’s experience of anxiety while implementing time-out and its impact on time-out implementation. Given that time-out can elicit temporary increases in both child and caregiver distress, especially when it is first practiced, it is possible that therapists similarly experience an increase in anxiety and distress (hereafter referred to as anxious distress) when teaching time-out in session, potentially leading to avoidance of using this important skill. Children’s emotions can become heightened, crying and screaming when time-out is implemented for the first time, eliciting an urge to end the time-out in order to appease the dysregulated child. Because delivering time-out can induce temporary anxiety for the child, caregiver, and therapist, it is an ideal intervention through which to study the role of therapist anxiety in implementation and potential maladaptive avoidance [14].

This study is the first attempt to empirically examine whether therapist anxious distress leads to avoidance of teaching parents to use time-out, a core evidence-based intervention for children with externalizing disorders. This study will contribute to literature aiming to understanding mechanisms of therapist decision-making with respect to evidence-based practice implementation in children’s mental health. If therapist anxious distress is, in fact, causing therapists to avoid using time-out and to deprive parents of an important strategy in mitigating their children’s disruptive behavioral symptoms, results will have important implication for training therapists and improving the quality of care. Using a clinical vignette-based survey design, our primary aim was to identify whether therapists experience anxious distress when implementing time-out in session and whether that anxious distress leads to avoidance of time-out. We hypothesized that anxious distress would rise as time-out progresses and that higher levels of therapist anxious distress would lead to higher likelihood of avoiding time-out. Exploratory analyses will identify predictors of time-out avoidance by examining whether time-out avoidance varies as a function of client factors (a history of maltreatment vs no history of maltreatment) and therapist professional factors (i.e., BPT training, years of practice).

## Methods

### Participants

Participants (*N* = 198) included mental health professionals who had seen a child or adolescent client up to 2 months prior to completing the survey. Participants had the option to skip questions, or select N/A for certain questions. Due to these options, there was slight variability about which were included in each analysis (range 169–190 participants). The participants identified their primary work setting as being a private practice (34.5%) or community agency (31.6%). 89.9% of participants describe working with children as a major part of their work, with the remaining 10.1% describing it as a minor part of their work. Roughly 85.3% of recruited mental health professionals reported having a master’s degree and 61.8% reported prior BPT training. Participants reported using time-out with 49.9% of young children with behavioral disorders who present to their practices (*SD* = 32.38), with other interventions being more commonly used (e.g. positive parenting strategies, relaxation techniques, and feeling identification). Nine participants reported never using time-out. Reported average years of practice as a therapist was 9.6 years (*SD* = 8.6). See Table 1 for additional participant characteristics.

**Table 1:** Participant characteristics

	Frequency (%)	Mean (SD)
Age (*n* = 177)		36.12 (10.62)
Years practiced (including training; *n* = 176)		9.58 (8.57)
Gender (*n* = 178)		
Female	161 (90.4%)	
Male	13 (7.3%)	
Non-binary/third gender	4 (2.2%)	
Race (*n* = 172)		
White	129 (75.0%)	
Black/African American	21 (12.2%)	
Multiracial	12 (7.0%)	
Asian American/Pacific Islander	6 (3.5%)	
American Indian or Alaska Native	2 (1.2%)	
Other race	2 (1.2%)	
Ethnicity (Hispanic/Latinx; *n* = 174)	21 (12.1%)	
Highest degree obtained (*n* = 177)		
Bachelor’s	5 (2.8%)	
Master’s	151 (85.3%)	
Doctorate	21 (11.9%)	
Licensed (*n* = 177)		
Yes	118 (66.7%)	
No	27 (15.3%)	
In progress	32 (18.1%)	
Work setting (*n* = 177)		
Private practice	61 (34.5%)	
Community agency	58 (32.8%)	
Hospital	19 (10.7%)	
School	16 (9%)	
College/university	12 (6.8%)	
Other	11 (6.2%)	

### Procedures

Participants were recruited through emails sent to child and adolescent mental health professional listservs across the US and recruitment advertisements on child and adolescent mental health professional social media groups. Participants completed a 20-min anonymous Qualtrics survey in which they were eligible to consent to the study if they reported that they had seen a child or adolescent client in the last two months. Upon consent, participants were randomly assigned to one of two versions of a three-part clinical vignette in which the reason for a family referral to treatment was varied: (1) a family was referred to treatment due to child externalizing behaviors or (2) a family was referred to treatment due to a history of maltreatment by the caregiver. Throughout both versions of the vignette the child presents with externalizing behaviors in session and the therapist coaches the caregiver to administer a time-out to the child in response to defiance. Participant received a $10 incentive upon survey completion. All study procedures were deemed exempt by the University of California Santa Barbara and National Association of Social Workers Institutional Review Boards.

#### Clinical Vignette

**Fig. 1:**
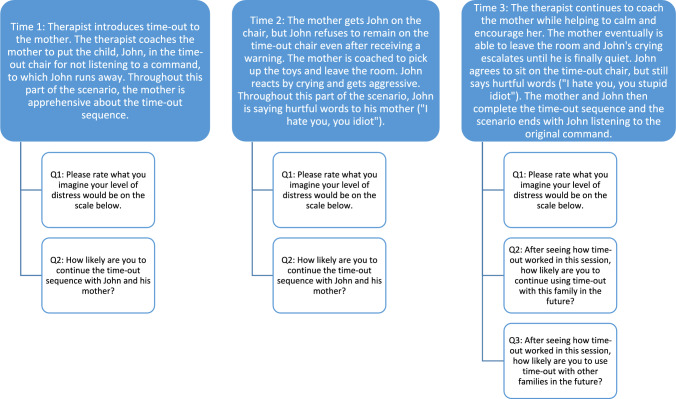
Flow of clinical scenario

Therapists read one of two versions of a clinical vignette of a typical early time-out session a therapist might deliver within treatment for disruptive or externalizing behaviors. The only difference between the versions was the reason for referral (i.e., the presence or absence of a history of maltreatment by the caregiver). The vignette was divided into three parts (hereafter referred to as “time points”). Throughout the three-part clinical vignette, survey respondents were asked to imagine that they were coaching the caregiver in the delivery of time-out and to rate their personal level of distress and avoidance (likelihood of abandoning the time-out or not delivering it in the future) if the situation were occurring. The first portion (“time one”) of the three-part vignette described a scenario in which the therapist introduces time-out to the caregiver by coaching the caregiver to bring the child to the time-out chair because the child ignored the caregiver’s command. During this part of the vignette, the caregiver seems hesitant and resistant to utilizing time-out with her child and the child is evading his mother’s attempts to put him in the time-out chair. Next (“time two”), the caregiver guides the child to the time-out chair and the child’s disruptive behaviors worsen. The caregiver is later coached to leave the room with the toys. The child’s disruptive behaviors escalate further, and he prevents his mother from leaving the room. In the final part of the clinical vignette (“time three”), the caregiver leaves the room, and the child continues to cry and plead, but eventually stops. The caregiver is then coached to continue with the time-out sequence and the vignette ends with the child listening and following directions, despite being upset. See Fig. 1 for a detailed flow chart of the clinical vignette.

### Measures

#### Participant Characteristics

Participants were asked to provide demographic information, including age, gender, race, ethnicity, education, and employment. Employment information included items that inquired about the mental health professionals’ training, years practiced, employment setting, populations served, and therapeutic strategies utilized.

#### Anxious Distress During Time-Out Implementation

At each time point of the clinical vignette, after the scenario was presented, participants were instructed to imagine themselves as the therapist and asked to rate their level of anxious distress at this point in the clinical encounter on an 11-point Likert-type scale from 0 (*totally relaxed*) to 10 (*highest anxiety/distress that you have ever felt*).

#### Avoidance of Time-Out

Avoidance was measured at each time point. Avoidance during the first two time points of the three-part vignette was measured by asking participants to rate the likelihood that they would continue the time-out sequence with the caregiver and child on a 7-point Likert-type scale from 1 (*not at all likely*) to 7 (*extremely likely*). Therapists also had the option to select “N/A (*I would not have chosen to use time-out with this family because it is not appropriate*)”. Avoidance at time three was measured by asking the participant first to rate the likelihood that they would continue to using time-out with the current family in the future and then the likelihood that they would use time-out with other families in the future on a 7-point Likert-type scale from 1 (*not at all likely*) to 7 (*extremely likely*). Higher likelihood of continuing with the time-out sequence indicated lower levels of avoidance.

#### Time-Out Administration Challenges

To evaluate specific challenges to delivering time-out, participants were asked to describe these challenges in an open-ended item after reading the clinical vignette.

### Data Analytic Plan

A mixed method design was utilized, in which both quantitative and qualitative data were collected simultaneously. Qualitative evaluation was secondary to the quantitative assessment (QUAN + qual; [36]). That is, quantitative analyses were utilized to understand therapist distress and how it relates to avoidance of time-out. Qualitative analyses were then used to explore the ways in which anxious distress and avoidance were identified and reported by therapists when describing challenges to delivering time-out through coded open-ended responses.

#### Quantitative Data Analysis

Descriptive statistics, repeated measures ANOVA, and logistic regression models were run to understand differences in reported anxious distress and avoidance at each time point of the three-part clinical scenario and with future families. Avoidance was dichotomized and analyzed using logistic regression models to examine differences among therapists endorsing no avoidance on the Likert scale and therapists endorsing at least some avoidance across time points and with future families. Therapists indicating N/A (*I would not have chosen to use time-out with this family because it 1 is not appropriate*) were excluded from avoidance analyses. We also ran all models categorizing therapists that reported N/A as demonstrating *at least some avoidance* and the models revealed the same results. Linear regression models were also run with avoidance measured on a continuous scale in which therapists rated their likelihood of continuing the time-out sequence at each time point. For interpretation purposes, avoidance scores were reverse scored so that higher scores indicated greater avoidance, such that 0 = low avoidance and 6 = high avoidance. Logistic regression analyses were then used to examine predictors of time-out use with future families. Exploratory bivariate correlations were run to investigate associations between previous use of time-out with young children with disruptive behaviors and anxiety and avoidance at each time point.

#### Qualitative Data Analysis

Of the 198 therapists who responded to the survey, 153 expanded on challenges to implementing time-out with children with disruptive behaviors. Open-ended responses were coded following recommendations for qualitative data analyses in implementation science described by Palinkas et al. [36], and the National Cancer Institute [37]. A coding team of two coders read all open-ended responses and developed a coding manual that included relevant codes. Coders then analyzed the open-ended responses to determine if they met the previously established codes by assigning the responses with a 0 (*No*) or 1 (*Yes*) for each code. The authorship team then reviewed the codes and conducted further thematic analysis to identify themes.

## Results

### Anxious Distress and Avoidance over Time

**Fig. 2:**
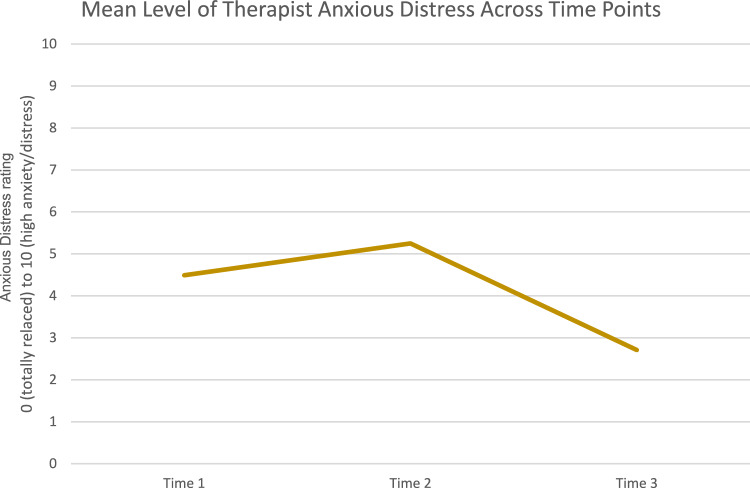
Therapist levels of anxious distress across the three time points

On average, therapists reported the highest levels of anxious distress at time 2 of the scenario (*M* = 5.25; *SD* = 1.88). Levels of therapist anxious distress were lower at time 1 (*M* = 4.49; *SD* = 1.88) and were the lowest, on average, at time 3 (*M* = 2.71; *SD* = 1.81). A repeated measures ANOVA with a Greenhouse–Geisser correction indicated that therapist-reported levels of anxious distress differed statistically across the three time points of the clinical vignette (*F*(1.638, 307.984) = 212.266, *p* < 0.001). Post hoc analysis with a Bonferroni adjustment demonstrated that anxious distress ratings significantly differed between time 1 and time 2 (0.762, *p* < 0.001), time 1 and time 3 (− 1.778, *p* < 0.001), and time 2 and time 3 (− 2.540, *p* < 0.001). See Fig. 2 for a visualization of therapist reported anxious distress across the three time points.

**Fig. 3:**
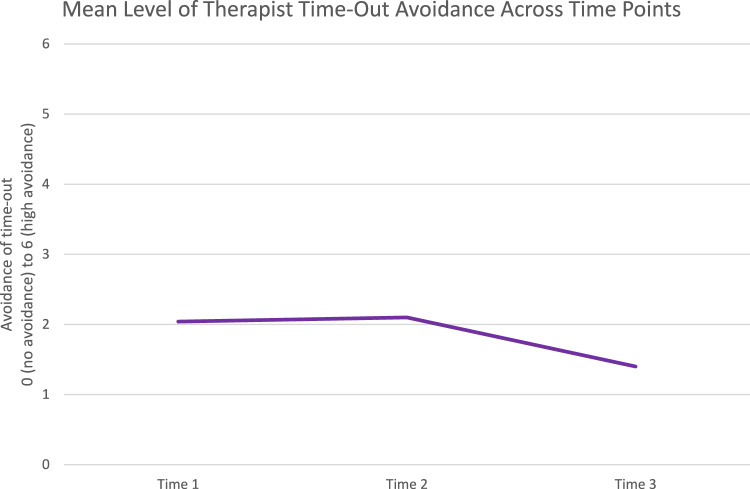
Therapist levels of time-out avoidance across the three time points

At time 1, 24 therapists indicated that they would not have chosen to use time-out at all because they perceived it as inappropriate. At time 2, 26 therapists indicated that they would not have delivered time-out. Of those that did report time-out avoidance levels on the Likert-type scale, reverse-coded time-out avoidance levels ranged from 0 (low avoidance) to 6 (high avoidance) at each timepoint, and were similar across time 1 (*M* = 2.04; *SD* = 1.92) and time 2 (*M* = 2.10; *SD* = 2.00). Time-out avoidance was lowest at time 3 of the three-part scenario (*M* = 1.40; *SD* = 1.72). Repeated measures ANOVA showed that therapist reported levels of time-out avoidance with the family in the clinical scenario across the three time points differed significantly (*F*(2, 320) = 32.579, *p* < 0.001). The difference across times 1 and 2 was not significant (0.062, *p* = 1.000). The difference in time-out avoidance was significant across time 1 and time 3 (− 0.640, *p* < 0.001), and time 2 and time 3 (− 0.702, *p* < 0.001). Figure 3 demonstrates therapist reported avoidance across the three time points.

With respect to dichotomized avoidance, at time 1, 44 therapists (25.3%) were *not likely to avoid* time-out at all and 130 therapists (74.7%) reported *at least some avoidance*. At time 2, 47 therapists (27.8%) were *not likely to avoid* time-out and 122 therapists (72.2%) reported *at least some avoidance*. At time 3, 72 therapists (37.9%) were *not likely to avoid* using time-out with the same family and other families in the future and 118 therapists (62.1%) reported *at least some avoidance* using time-out with the same family and other families in the future.

#### Relationship Between Anxious Distress and Time-Out Avoidance

Table 2 shows results from the logistic regression models that examined the relationship between levels of anxious distress and time-out avoidance (0 = *Not likely to avoid*; 1 = *At least some avoidance*) at each time point during the three-part clinical scenario. Time 1 anxious distress did not relate to time-out avoidance at time 1 (OR 0.988; 95%CI [0.820, 1.191]), nor did time 2 anxious distress relate to time 2 time-out avoidance (OR 1.017; 95%CI [0.846, 1.222]). However, as anxious distress increased at time 3, therapists were 1.366 times more likely to endorse time-out avoidance when delivering time-out with the same family in the vignette (95%CI [1.135, 1.645]) and 1.445 times more likely to endorse time-out avoidance when delivering time-out with future families (95%CI [1.192, 1.751]).

**Table 2:** Results of binary logistic regression analyses examining time-out avoidance across time points

	*B*	*SE*	Wald	95% CI		*p*
				LL	UP	
Time 1						
Intercept	1.135	0.458	6.138			0.013
Level of anxious distress	− 0.012	0.095	0.015	0.820	1.191	0.902
Time 2						
Intercept	0.867	0.514	2.845			0.092
Level of anxious distress	0.017	0.094	0.032	0.846	1.222	0.859
Time 3 (current family)						
Intercept	− 0.314	0.278	1.270			0.260
Level of anxious distress	0.312	0.095	10.912	1.135	1.645	0.001
Time 3 (future families)						
Intercept	− 0.448	0.283	2.518			0.113
Level of anxious distress	0.368	0.098	14.073	1.192	1.751	0.000

Table 3 shows the results from the linear regression models that tested the relationship between therapist-reported anxious distress and time-out avoidance, when avoidance was treated continuously. Consistent with the logistic regression models, time 1 anxious distress did not relate to time-out avoidance at time 1 (*B* = − 0.094, *p* = 0.264), nor did time 2 anxious distress relate to time 2 time-out avoidance (*B* = 0.052, *p* = 0.554). However, higher time 3 anxious distress was associated with greater therapist time-out avoidance when asked about future use of time-out with the same family in the vignette (*B* = 0.423, *p* < 0.001, *R*^*2*^ = 0.13, a medium effect) and when delivering time-out with future families (*B* = 0.4, *p* < 0.001, *R*^*2*^ = 0.12, a medium effect).

**Table 3:** Results of linear regression analyses examining time-out avoidance across time points

	*B*	*SE*	*p*
Time 1			
Intercept	2.658	0.402	0.000
Level of anxious distress	− 0.094	0.084	0.264
Time 2			
Intercept	1.938	0.481	0.000
Level of anxious distress	0.052	0.087	0.554
Time 3 (current family)			
Intercept	0.774	0.260	0.003
Level of anxious distress	0.423	0.080	0.000
Time 3 (future families)			
Intercept	0.807	0.255	0.002
Level of anxious distress	0.400	0.078	0.000

#### Predictors of Future Time-Out Use

Table 4 presents the results from the logistic regression analyses used to examine predictors of time-out use with future families (0 = *Not likely to avoid*; 1 = *At least some avoidance*). Prior BPT training was associated with less time-out avoidance with future families (*OR* 0.260, 95%CI [0.128, 0.530]). Presence or absence of a history of maltreatment in the clinical vignette (95%CI [0.598, 2.155]) and number of year practicing (95%CI [0.980, 1.057]) did not relate to therapist reported use of time-out with future families.

**Table 4:** Results of binary logistic regression analyses predicting time-out avoidance with future families

	*B*	*SE*	Wald	95% CI		*p*
				LL	UP	
Intercept	1.115	0.361	9.528			0.002
Years practiced	0.017	0.019	0.808	0.980	1.057	0.369
Child maltreatment (Yes)	0.127	0.327	0.152	0.598	2.155	0.697
Prior BPT (Yes)	− 1.345	0.363	13.770	0.128	0.530	0.000

#### Previous Therapist Time-Out Use and Current Anxious Distress and Avoidance

Table 5 presents bivariate correlations used to investigate the relationship between previous time-out use by therapists in their practices and anxious distress and avoidance in the current vignette. The percent of young children with disruptive behaviors with whom therapists report using time-out was significantly correlated with anxious distress at time 3, and avoidance at each timepoint. Use of time-out with a higher percentage of clients was associated with lower anxious distress at time 3, and lower avoidance at each timepoint.

**Table 5:** Correlation matrix

	1	2	3	4	5	6
1. Use of time-out^a^	–					
2. Time 1 anxious distress	0.035	–				
3. Time 2 anxious distress	− 0.092	0.769**	–			
4. Time 3 anxious distress	− 0.237**	0.420**	0.475**	–		
5. Time 1 avoidance	− 0.458**	− 0.050	− 0.044	0.165*	–	
6. Time 2 avoidance	− 0.450**	− 0.034	− 0.056	0.132	0.777**	–
7. Time 3 avoidance	− 0.568**	− 0.034	0.072	0.356**	0.726**	0.730**

^a^% of clients (young children with disruptive behaviors) with whom therapist uses time-out in current practice

**Correlation is significant at the 0.01 level

*Correlation is significant at the 0.05 level

### Qualitative Results

Therapists described multiple challenges to delivering time-out with youth with disruptive behaviors. Challenges reported fell into four primary themes, including time-out eliciting *too much distress in the parent or child*, time-out eliciting *anxiety/distress in the therapist*, time-out being *difficult to implement with consistency and follow-through*, and time-out being *ineffective*. Themes are described below and additional illustrative quotes are found in Table 6.

**Table 6:** Themes and illustrative quotes (challenges to delivering time-out)

Theme	Quote
Parent or child distress	
Some children are also so neurologically dysregulated that they need a lot of helping learning to control themselves enough to go to time-out	
Making sure they can tolerate the distress	
Managing both distress of parents and the distress of the child	
Big feelings or outbursts in children	
Parents having the distress tolerance to manage the disruptive behavior	
Therapist anxiety/distress	
Fear of escalating self-harming or abusive behaviors	
Fear of triggering feelings of abandonment in child	
Dealing with the distress it causes the child and parents	
Difficult to implement	
Caregivers implementing the time-out incorrectly	
Getting them to remain in the time-out chair	
Establishing enough parental buy-in	
Ineffective	
It could be ineffective, harm the therapeutic relationship, and make the situation worse	
It’s a terrible intervention that is not effective	
This is not an effective strategy for this population especially because disruptive behaviors are primarily evidence of the child communicating an unmet need	

Many of the responses provided by therapists highlighted a concern about *parent or child distress*, with therapists explaining that time-out is “distressing to children, distressing to parents.” Regarding caregiver distress specifically, therapists stated that “parents not being able to stay regulated and calm,” and “parent fear” are both challenges to implementing time-out. Regarding children, they described that “time-out provokes more distress than many young children can bear.” In addition to frequently reporting that one of the primary challenges to delivering time-out is the caregivers’ and child’s distress, respondents described *their (the therapists’) own distress* as an obstacle to implementation. One described it challenging to “coach the parent in a calm, confident manner,” and others described time-out as “causing serious distress for all parties involved.” Another explained that they struggled to “tolerate how much distress [time-out] would cause in the clients and their parents.”

Therapists also reported that time-out is *difficult to implement*, due to challenges to “parents maintaining consistency and committing the time to proper use, [and] the parents’ ability to follow through in an appropriate manner.” Many therapists described challenges to parental consistency and practical difficulties to getting the child into time-out, and difficulties “convincing parents who have tried time-out before that it could be more effective if it is practiced differently.” Therapists also described their own beliefs about time-out being *ineffective*, reporting, for example, that “it’s not effective for long term behavioral change and leads to power struggles between parents and children.”

## Discussion

This study aimed to evaluate therapist anxious distress implementing time-out and avoidance of implementing time-out, and to further understand challenges to its implementation. Results of this study indicate that, as was hypothesized, anxious distress increased as the time-out progressed, but decreased at its resolution. Regarding whether therapist anxious distress is associated with time-out avoidance, findings both confirmed and disconfirmed aspects of this hypothesis. Higher anxious distress during the time-out delivery (at time 2) did not increase the likelihood of abandoning the time-out midway, in contrast with the study’s hypothesis. However, higher anxious distress at the time-out’s resolution (time 3) predicted a higher likelihood of avoiding time-out in the future with both the same client and with other clients.

These findings are aligned with research indicating that therapist anxiety contributes to drift from evidence-based interventions [17], and adds implementation insights related to working with young children to literature that has primarily focused on interventions for adults. The findings also suggest that therapist anxious distress may not lead to premature termination of a time-out midway through, but that if the therapist’s anxious distress does not adequately resolve at the time-out’s end, their decisions about future use of the EBI may be impacted. Interestingly, the clinical vignette ends with a successful time-out, in which the child complies with the parent’s request. Despite the successful resolution, 62.1% of therapists reported at least some avoidance of using time-out in the future. Although this likelihood of avoidance decreased from time points 1 and 2 of the vignette, it remains high, suggesting that even experiencing a successful time-out delivery and resolution may not mitigate the impact a therapist’s anxious distress has on their future treatment decisions. It would be interesting to know what would decrease the likelihood of avoidance, if not a successful in-session time-out delivery. Given that prior BPT training was associated with lower avoidance in the current study, it is possible that greater understanding of the purpose of time-out, its theoretical justification, or more experiences of using it successfully may mitigate anxious distress or the impulse to abandon the intervention. In fact, the associations found in exploratory analyses between higher therapist use of time-out in their practices and reported lower anxious distress and avoidance in the clinical vignettes provides initial evidence that more experience with the procedure may mitigate the impulse to abandon it.

Qualitative findings expanded on quantitative results in this study, providing insight into particular challenges therapists experience that may contribute to their own anxious distress and subsequent avoidance of delivering time-out. Specifically, therapists described their own fears and discomfort delivering time-out, with particular focus on the distress it could cause the caregiver or child. They perceived the intervention to be ineffective and difficult to implement in practical ways, as well. These findings further indicate that a greater understanding of the procedure, including how to implement it successfully, its theoretical justification, and outcomes for families and children, may decrease therapist distress and increase effective implementation. These findings also point to the importance of preparing clinicians in advance for how to emotionally regulate common affective responses to implementing time-out. Future research on implementation strategies could investigate the impact of both didactic training addressing theory, justification, and procedures, and experiential training on therapist perceptions and uptake of time-out.

Given that therapist anxious distress at time point 3 increased the likelihood of avoiding time-out in the future, addressing therapist anxious distress during and after an intervention may improve clinical decision-making. Therapists should potentially be trained specifically to manage their own anxious distress during treatment. In fact, developing implementation interventions that address therapist distress, such as helping therapists learn to manage their own anxious reactions, has been recommended [14, 38]. A recent study found that 12 therapists who underwent experiential training reported improved attitudes towards exposure, one underused EBI [39]. While this intervention did not specifically evaluate therapist anxiety, experiential training may have decreased therapist anxiety, leading to more positive attitudes towards the practice. Therapist anxiety sensitivity has also been found to predict lower clinical proficiency in delivering exposure therapy [40]. In Harned and colleagues’ study, therapists who reported higher fear of anxious sensations at baseline demonstrated lower proficiency in delivering exposure therapy after receiving training in it. It was hypothesized that these therapists may have engaged in more anxiety-mitigating strategies during delivery, in an attempt to alleviate their and their client’s discomfort. Unfortunately, these strategies render the intervention less effective. Therapist concerns referenced in open-ended responses in the current study, describing worry about the client’s or one’s own distress, may reflect a similar fear of eliciting temporarily distressing sensations. Therapists whose anxious distress contributes to time-out avoidance, as well as those whose anxiety-sensitivity contributes to hesitant and less effective service-delivery may benefit from trainings that specifically teach them to manage their own anxiety during treatment delivery.

The present study was limited in that it relied on therapist self-report. Outcomes were evaluated based on therapist reports about hypothetical behaviors and avoidance, which may or may not closely approximate their in-session actions. It may be challenging to accurately measure avoidance, given that practitioners may be hesitant to admit that they would avoid engaging in a best-practice, particularly if their avoidance is based on anxious distress. For this reason, we measured intention to use time-out, which we believed clinicians would report more honestly, and used the reverse score as a measure of avoidance. Strength of intention to use a practice is frequently used in implementation research [41]. Previous research has found that strength of reported intention to use an evidence-based practice does predict subsequent use of that practice. In one study, teachers who reported a strong intention to use a practice were 5.2 times more likely to actually use the practice than teachers who had weak intentions to use them [42]. Additionally, similar ratings of intention to use a practice have been used in previous studies, and such scales have been found to be predictive of subsequent clinician behavior (i.e. use of specified EBI) [43, 44]. Despite these efforts to measure avoidance as accurately as possible, future research would benefit from live observations of time-out delivery, or from other methods of assessing in-session behavior, such as review of clinical documentation or ecological momentary assessment (EMA), which could capture self-reports throughout the week as therapists go about delivering services [45]. EMA gathered during time-out delivery in practice would provide real-time insight into therapists’ anxious distress and desire to abandon implementation while circumventing the inaccuracy or recall or imagined vignettes.

Additional limitations include that this study did not gather information about therapist current caseload; although the study asked therapists to consider a specific vignette-based case, it is possible that caseload or setting may impact decisions about whether to use time-out with clients or not. A final limitation in this study was the potential conflation of the terms anxiety and distress when asking therapists to report how they were feeling during the vignette. Although the scale went from “totally relaxed” to “highest anxiety/distress that you have ever felt,” it is possible that therapists interpreted this scale in different ways, with some feeling worried about how the time-out would go, and others finding it upsetting for other reasons. At the same time, distress is often used as a term in the anxiety treatment literature to assess perceptions of anxiety and distress in given situations, as in Subjective Units of Distress (SUDS). It would be beneficial to understand more about the specific physiological sensations and cognitions therapists envisioned when reporting on their anxious distress, to confirm the cause of any subsequent avoidance. By collecting qualitative responses, we have begun to disentangle specific therapist concerns and reactions to delivering time-out, however the nature of their anxious distress should be further investigated. Despite its limitations, this study provides initial insight into an important factor shaping the implementation of an important EBI for young children; teaching caregivers to safely and effectively use time-out.

Despite limitations, this article contributes to a more robust understanding of treatment fidelity and clinical decision-making. Prior research on how therapist factors impact EBI implementation has focused on therapist attitudes, beliefs, and self-efficacy. Few studies have specifically investigated therapist subjective reports of experiencing anxious distress while delivering an intervention, and how this may impact their decision-making. Since time-out can be a particularly challenging intervention to implement and teach, and is currently the focus of much public discourse in the parenting community [27, 32], it is a useful EBI to investigate. While researchers have attempted to understand controversy about time-out [27, 46], this is the first to explore therapist subjective experiences of anxious distress while delivering the intervention. Attending to systemic factors that impact the implementation of EBIs, such as training, leadership, and agency culture, as well as client factors, is important, considering therapist anxious distress may further support the implementation and delivery of EBIs, and help ensure that clients are receiving clinically-indicated evidence-based care. It will be important to develop implementation interventions specifically addressing therapist anxious distress in the future.

## Summary

Many leading evidence-based interventions (EBIs) in psychosocial treatment are complex. One understudied factor that contributes to the underuse of complex EBIs is a therapist’s own anxious distress, particularly regarding EBI components that elicit temporary distress in the client (e.g., exposure therapy for anxiety, time-out for externalizing disorders). Therapist anxious distress may lead to avoidance of the EBI, impeding implementation. Therapist anxious distress when delivering child mental health treatment may contribute to the underuse of some evidence-based interventions, such as time-out for children with disruptive behaviors. This study investigated therapist anxious distress and avoidance related to coaching caregivers to use time-out using a three-part, vignette-based survey design. Therapists (n = 198) read a vignette describing a time-out. At three different points throughout the vignette, therapists reported on their personal anxious distress via Subjective Units of Distress (SUDS) and their likelihood of discontinuing the implementation of time-out in session. Binomial logistic regression analyses examined the relationship between self-reported anxious distress at all 3 time points and likelihood of avoiding time-out to test the hypothesis that elevated anxious distress would be related to higher likelihood of avoiding time-out. Therapists were also asked about challenges to delivering time-out. Open-ended responses were thematically analyzed to further understand avoidance of time-out. Therapists reported moderate levels of anxiety at time points 1 (M = 4.49; SD = 1.88) and 2 (M = 5.25; SD = 1.88) and lower levels of anxiety at time 3 when the time-out had resolved (M = 2.71; SD = 1.81). Most therapists endorsed at least some avoidance of (likelihood of discontinuing) time-out at time 1 (n = 130, 74.7%) time 2 (n = 122, 72.2%), and time 3 (n = 118, 62.1%). Binomial logistic regression analyses at each time point indicated that increased anxious distress corresponded with an increased probability of avoiding time-out delivery only at time point 3 with the current family in the future (OR 1.366; 95%CI [1.135, 1.645]) and other families in the future (OR 1.445; 95%CI [1.192, 1.751]). Qualitative reports expanded on challenges therapists perceived to implementing time-out, including their own distress, the child or parents’ distress, difficulties with parental consistency, and perceiving time-out as ineffective. Findings suggest the potential utility of directly addressing therapist anxiety when providing training and consultation in certain complex EBIs.

## Data Availability

The open-ended responses generated and analyzed during the current study are not publicly available to protect the identities of participants, but are available from the corresponding author on reasonable request.
